# Performance of the bioLytical Multiplex HIV 1/2 and Syphilis rapid test on serum in a laboratory evaluation for syphilis

**DOI:** 10.1128/spectrum.00132-25

**Published:** 2025-05-19

**Authors:** Anastasia Eliopoulos, Beatrice Rivera, Emily McCullough, Ameeta E. Singh, Jennifer Gratrix, Graham Tipples, Hong Yuan Zhou, Rick Galli, Sean B. Rourke, Kevin Fonseca

**Affiliations:** 1 Public Health Laboratory, Alberta Precision Laboratories 590576 , Calgary, Alberta, Canada; 2 Public Health Laboratory, Alberta Precision Laboratories, Edmonton, Alberta, Canada; 3 Department of Medicine, University of Alberta 215465, Edmonton, Alberta, Canada; 4 STI Services, Alberta Health Services 3146, Edmonton, Alberta, Canada; 5 Department of Laboratory Medicine and Pathology, University of Alberta 536883, Edmonton, Alberta, Canada; 6 Reach Nexus, MAP Centre for Urban Health Solutions, St. Michael’s Hospital, Unity Health Toronto, Toronto, Canada; 7 MAP Centre for Urban Health Solutions, Unity Health Toronto 508783, Toronto, Ontario, Canada; 8 Department of Psychiatry, University of Toronto 7938, Toronto, Ontario, Canada; 9 Department of Microbiology, Immunology and Infectious Diseases, University of Calgary 2129, Calgary, Alberta, Canada; University of Chicago, Chicago, Illinois, USA

**Keywords:** syphilis, rapid test, point-of-care test, serum, rapid plasma reagin, treponemal antibody, non-treponemal antibody, signal/cutoff ratio

## Abstract

Syphilis, a sexually transmitted infection, is resurging with large outbreaks worldwide. In pregnant females, vertical transmission can result in stillbirths and serious sequelae for the fetus. Syphilis serology is primarily used to make the diagnosis, but the testing platforms and confirmatory tests are often centralized; therefore, the time to a result is usually more than 2 days. Most affected populations in the current outbreaks can be transient and easily lost to follow up. Therefore, rapid testing can assist with timely detection, treatment, and linkage to care. We evaluated the performance of the INSTI Multiplex HIV-1/2 Syphilis rapid test, using a large panel of negative and confirmed positive sera with varying rapid plasma reagin (RPR) titers. Sera with RPR titers of 1:8 and greater consistently gave a positive rapid test result (93.8%–100%; 95% CI: 82.5%–100%), whereas samples with RPR titers of 1:2 and lower were often negative (7.6%–56.5%). Rapid test positive samples generally had a syphilis CMIA S/CO ratio of 19.78 and higher, although there was no specific ratio that separated all positive from negative rapid results. In infectious syphilis, RPR titers are often 1:8 and greater and should be reliably detected with this rapid test (accuracy 97.2%), thereby supporting immediate treatment and linkage to care with a high level of confidence. Additionally, a result is available within minutes and therefore could be included on acute care STAT laboratory test menus while the individual is an in-patient or awaiting assessment in the Emergency Department.

**IMPORTANCE:**

We examined the performance of the syphilis antibody component of a rapid multiplex device and found that sera with RPR titers of 1:8 and greater, often found in infectious syphilis, were highly likely to test positive. This device would be suitable for providing STAT syphilis serology testing for suspect cases waiting in the Emergency Department or as inpatients. Positive results can support immediate treatment and linkage to care, especially for pregnant females and transient persons who are often lost to follow up.

## INTRODUCTION

Rates of both congenital and infectious syphilis infections have seen significant increases in Canada, USA, and worldwide (1). In Canada, the national rate of infectious syphilis increased from 5.1 per 100,000 population in 2011 to 36.1 per 100,000 in 2022 (2), with the Prairie provinces recording a relative increase of around 105% since 2018, also coupled with an increase in congenital syphilis cases (3). There have been two prior outbreaks of syphilis in Alberta, from 1981 to 1987 (4) with over 1,089 cases and a smaller outbreak from 2004 to 2006 with five congenital cases recorded (5). However, between 2015 and 2023, there was at least a 10-fold increase in the number of infections, with over 17,000 cases documented in adolescents and adults. As a result, the Government of Alberta declared a syphilis outbreak in 2019 reporting 2,331 cases of syphilis that year, and for 2022 and 2023, 6,033 cases were reported. The increased burden of syphilis infections in the community has, in turn, led to an increase in the cases of congenital infections from four cases in 2016 to over 340 cases from 2015 to March 2024, resulting in at least 60 stillbirths, which has been attributed to a 10-fold increase in infectious rates in females of childbearing years (6, 7).

Identification of syphilis cases is primarily through serologic testing because the manifestations of this infection can range from inapparent to a wide spectrum of neurological, disseminated or localized rash, and other diverse presentations (8). In a new syphilis infection, the affected individual produces both treponemal and non-treponemal antibodies, which can be detected by different serology tests. Treponemal antibodies, directed to the surface membrane proteins of *Treponema pallidum,* persist for many years even after effective treatment, unless the person is treated very early in the course of the infection (8). Examples of treponemal assays are the *Treponema pallidum* chemiluminescent microparticle immunoassays (CMIA), *T. pallidum* particle agglutination assay (TPPA), fluorescent treponemal absorbed (FTA-Abs), and *T. pallidum* immobilization assay (TPI). In contrast, non-treponemal antibodies are formed in response to the cardiolipin components of the host cells and the spirochete cell membrane (9) and are often determined by the rapid plasma reagin (RPR) card and Venereal Diseases Research Laboratory (VDRL) tests (10). These antibodies are elevated in the acute, secondary, and early latent phases of syphilis and show a significant reduction in RPR titers after treatment, and therefore, they are used to monitor the effectiveness of therapy. Infrequently, in the untreated individual, these titers can also decline, but this reduction occurs more slowly (8). In reinfections, the non-treponemal antibodies measured as RPR titers show an increase, whereas residual treponemal antibodies from a prior infection can also show an increase, but routinely are not reported quantitatively (11). Therefore, the RPR titers, in conjunction with the clinical history, are used to clinically stage each case, which determines the length of treatment and follow-up timeframe for their sexual contacts.

Serology testing is often performed at central laboratories, and the results can take from a few days, for a negative result, to 1 week or more for a positive result, in part because the samples have to be collected and transported to the testing laboratory. Here, the samples are tested on automated platforms, often using a reverse testing algorithm that incorporates a combination of screening and confirmatory assays before negative and confirmed positive results can be reported. Additionally, once positive results are notified to public health authorities, additional time is needed to locate these cases and their sexual contacts for treatment. In transient populations, where much of the transmission occurs, many of these cases are lost to follow up before the result is available (12). Therefore, earlier detection with rapid point-of-care tests (POCT) can potentially result in earlier treatment of the index case and follow-up of their contacts. For pregnant females in particular, the treatment of the mother and unborn child requires immediate linkage to specialized services to assess the duration of treatment and ongoing follow-ups for the potential sequelae that could arise for the fetus.

Although there are a number of syphilis POCT serology tests in use globally, in the United States just two, namely, the Syphilis Heath Check (Diagnostic Direct LLC, New Jersey, USA) and Dual Path Platform (DPP) HIV-Syphilis (Chembio Diagnostics Systems Inc, New York, USA) are Food and Drug Administration cleared and Clinical Laboratory Improvement Amendments (CLIA) waived for syphilis antibody detection (10). Neither of these two kits has Health Canada approval at the time of writing and cannot be used for routine diagnostic testing in Canada. In clinical trials, their sensitivities ranged from 50% to 95% and specificities from 88.7% to 100%, using the TPPA as the gold standard, which detects just treponemal antibodies (13). The Chembio Dual Path Platform (DPP) was evaluated in a Canadian study in Arctic communities (14), where there is a large syphilis outbreak ongoing and who experience poor access to health services. A key finding is that the authors reported an overall sensitivity of 94% for the non-treponemal component, rising to almost 100% when the RPR titer was at least 1:8 and therefore suggestive of an infectious syphilis case. A minor drawback is the digital reader used in the assay can take up to 20 min to yield a result (14). Nonetheless, this study shows the value of a POCT assay to identify potential infectious cases in a highly mobile group and treat them while they were still accessible.

The 2022 point-of-care test for syphilis and HIV (PoSH) study conducted by Singh et al*.* (15) compared the performance of two POCT devices, the INSTI Multiplex HIV 1/2 syphilis antibody test (bioLytical Laboratories, Inc., Richmond, British Columbia), and Multiplo TP/HIV test (MedMira Inc, Halifax, Nova Scotia), which detect antibodies to both syphilis and HIV 1 and 2, using a fingerstick blood sample collected from a high-risk and transient population. In addition, standard syphilis and HIV serology were also performed as the reference comparators. Both these Canadian-manufactured kits provided a result within 5 min or less from the addition of the sample into the test cassette. After evaluating the results from 1,364 encounters, there were 377 positive POCT results from the INSTI Multiplex and 425 from the Multiplo POCT for the syphilis component of the assays, compared with 489 positive results by standard syphilis serology. More than half (53.6%) of the study participants were previously diagnosed with syphilis, but for the remainder of newly diagnosed patients, except for one case lost to follow up, the rest were treated either during the time of testing or between 4 and 8 days after the POCT diagnosis. A significant finding was that an RPR titer of 1:4 and greater increased the positive predictive value from 97% to 99.1%, which in turn allows for a positive POCT result, for both rapid tests, to be a strong indication to treat without waiting for further confirmatory testing. Although pre-treatment RPR titers of 1:8 and greater are imperfect at distinguishing early from late syphilis stages, they are a useful adjunct in prioritizing syphilis cases as infectious and hence the follow-up of partner notification (16). Likewise, a positive rapid syphilis result could be used to prioritize immediate treatment, especially in a person with no prior syphilis testing or with a previous negative result, if at risk for loss to follow up, such as those in rural or remote communities who are highly transient and difficult to reach through usual healthcare pathways.

In the province of Alberta, occupying an area of 661,848 km², the Public Health Laboratory (ProvLab) is part of a provincial network of clinical laboratories, performing diagnostic syphilis testing for the whole province at two centralized locations in two large urban cities. Consequently, all samples have to be transported to either of these two sites, which can take at least 1 or more days due to the geographical size of the province. For many years, our laboratory has provided oversight for decentralized rapid HIV testing in select acute care laboratories to assist with post-exposure prophylaxis management. Therefore, the availability of a Health Canada-licensed rapid syphilis kit provided the opportunity to make syphilis testing available at acute care laboratories located in areas with high rates of syphilis. As serum samples are routinely collected for standard syphilis, HIV testing, and other STAT tests, we aimed to assess the performance of the bioLytical INSTI Multiplex assay, licensed by Health Canada in March 2023 (17), using a large panel of characterized serum samples.

## MATERIALS AND METHODS

### Sample selection criteria

Serum samples sent to the Public Health Laboratory specifically for syphilis diagnostic testing were used in this study and were leftover aliquots, anonymized and unlinked with no personal identifiers. Most of the sera came from clinics specializing in sexually transmitted infections. Serum samples for the pregnant category of this evaluation were selected from those sent for routine prenatal infectious diseases testing, which includes syphilis as one of the six infectious conditions; these bloods are usually collected in the first trimester.

The syphilis negative samples were selected from a 3-month period prior to the evaluation commencing, whereas the positive samples, especially those with high RPR titers, were archived samples stored at −20°C. We attempted to exclude samples that were serologically confirmed for HIV antibodies from this study.

### Syphilis serology testing

This laboratory uses the reverse algorithm for syphilis testing and interpretation; therefore, samples were initially screened for IgM and IgG treponemal antibodies to TpN15, TpN17, and TpN47 recombinant antigens in the Abbott Alinity platform (Abbott GmbH, Weisbaden, Germany), which is a chemiluminescent microparticle immunoassay [CMIA]. Samples testing negative (signal/cutoff ratio [S/CO < 1.00]) are reported as not reactive, whereas sera that are reactive (S/CO > 1.0) are then tested in the Rapid Plasma Reagin (RPR, Macro-Vue Card test, [Becton, Dickinson, MD, USA]) to determine an RPR titer, expressed either as endpoint dilutions (dils) or titers. Samples from previously undiagnosed cases, with or without an RPR titer, were confirmed for treponemal antibodies in the *T. pallidum* particle agglutination assay (Fujirebio, PA, USA), whereas those previously confirmed as cases were not retested again in the TPPA, due to the persistence of treponemal antibodies, even after treatment. All assays were performed, and results were interpreted as recommended by the respective manufacturer. Antibody negative and positive samples were stored at −20°C until discarded or later archived as per the laboratory storage policy.

### Rapid test kit

The INSTI Multiplex HIV-1/2 Syphilis test (bioLytical Laboratories, Richmond, British Columbia, Canada), hereafter referred to as the INSTI multiplex or rapid test, is a single-use, flow-through qualitative immunoassay for the detection of antibodies to both HIV-1 and 2 and syphilis in serum, whole blood and plasma, licensed by Health Canada in March 2023 (17).

The antigens to the two agents are a combination of recombinant envelope proteins, namely, gp41 (HIV-1), gp36 (HIV-2), and a treponemal fusion protein to p17 and p47 envelope domains of *T. pallidum* (18). These two antigens, in addition to an IgG/IgM antibody indicator, are separately bound to a filtration membrane at three separate locations on the cassette membrane. The IgG/IgM spot also serves as an internal quality control to indicate there is sufficient total antibody produced by the subject, who, if infected, can mount a demonstrable antibody level to the two infectious agents. This test usually takes from 1 to 3 min to complete after the addition of 50 μL of the serum to the sample dilution reagent, which, after mixing, is poured into the test cassette. Visible blue spots, indicating the presence of a valid internal control and the presence of the syphilis and/or HIV antibody, usually appear immediately or within 30 s after the addition of the second antibody detection reagent, which is colored blue. Finally, a clearing reagent is added, which serves to reduce the background arising from the detection reagent, thereby making reading fainter dots easier, especially helpful when using whole blood or a fingerstick sample.

The testing and interpretation of results were performed as stated in the manufacturer’s package insert. The test and control dots were read as positive, negative, or indeterminate without reference to an external visual chart. Essentially, a positive result was the presence of a visible blue dot at the control and syphilis locations, and a negative result was just the presence of the internal control. The technologist performing the rapid assay was blinded to the standard syphilis serology results to prevent any bias in the reading of the INSTI multiplex assay.

Once all samples were tested, the syphilis results were reviewed by a second technologist, and any samples that yielded results significantly different from an expected result, previously noted in the PoSH study (15), were retested in the rapid assay. When necessary, the syphilis CMIA and RPR were rerun to verify the S/CO ratios or RPR titers were similar to the initial result. Only the results from the syphilis component of the INSTI multiplex were recorded for this evaluation.

Sensitivity, specificity, negative (NPV), and positive (PPV) predictive values and confidence intervals were determined using standard statistical equations available on Microsoft Excel version 2406 and Graph Prism version 9.2.

## RESULTS

Table 1 describes the demographics of the samples comprising the serum panel selected for just the evaluation of the syphilis component of the INSTI multiplex assay. The overall panel comprised 1,350 samples, 900 that were syphilis CMIA nonreactive and 450 that were CMIA reactive. The reactive samples were also confirmed by the TPPA, performed either on the panel sample or on one or more samples previously collected from that patient to confirm the diagnosis of syphilis.

**TABLE 1:** Distribution of syphilis-positive and -negative panel samples by age range, gender, and pregnancy status

Category	Sample demographics			Subtotal
	Age range (yr)	No. of females (no. pregnant)	No. of males	
CMIA nonreactive sera (S/CO < 1.0)	15–20	46 (1)	27	73
	21–30	200 (80)	109	309
	31–40	181 (92)	144	325
	41–50	55 (7)	52	107
	51–60	22 (0)	29	51
	>60	18 (0)	17	35
	Subtotal	522 (180)	378	900
CMIA reactive sera (S/CO >= 1.0)	15–20	17 (7)	5	22
	21–30	92 (38)	45	137
	31–40	87 (36)	89	176
	41–50	25 (8)	39	64
	51–60	8 (0)	24	32
	>60	2 (0)	17	19
	Subtotal	231 (89)	219	450
	Total	753 (269)	597	1,350

The 900 CMIA nonreactive samples were all collected from different individuals. Sera from the female category were further subgrouped into pregnant and non-pregnant female groups. Samples from pregnant females were specifically included, as they are known to be associated with false-positive serology results, and this evaluation was an opportunity to assess how often this phenomenon might occur in this specific population. Furthermore, as there are a significant number of congenital syphilis cases occurring during this outbreak, samples from the pregnant female population provide a further reason to assess the impact of this test. In the CMIA nonreactive panel, also inclusive of the pregnant female samples, we did not encounter any samples giving invalid results, namely the visual absence of the internal control dot. None of the 900 serum samples that were serologically negative for syphilis antibody in the standard serology assay gave a positive result in the INSTI multiplex kit (Table 1), including the female pregnant panel of 180 samples.

There were 450 syphilis-positive samples in the panel, defined by their reactivity in both the CMIA and TPPA to exclude biological false-positive samples (Table 2). These positive sera were further subdivided by varying RPR titers, together with the cumulative INSTI multiplex positive and negative results obtained at each titer. The highest RPR titers in this panel were from two cases, one male and the other female, both of whom recorded a titer of 1:4,096. Also included were syphilis-positive samples from 89 pregnant females, with the highest recorded RPR titer of 1:512 in one of these persons.

**TABLE 2:** Distribution of CMIA syphilis-positive samples by RPR titer, INSTI multiplex result, and S/CO ratio ranges

RPR titer	No.	INSTI positive result			INSTI negative result		CMIA S/CO ratio range (mean)
		No.	%	95% CI	No.	%	
Not reactive	66	5	7.6	2.9–16.9	61	92.4	1.19–18.32 (9.60)
1:1	24	8	33.3	17.8–53.4	16	66.7	8.41–21.83 (15.56)
1:2	40	23	57.5	42.2–71.5	17	42.5	9.11–23.37 (17.25)
1:4	30	25	83.3	65.9–93.1	5	16.7	7.90–23.12 (18.16)
1:8	48	45	93.8	82.5–98.5	3	62.5	4.73–24.57 (19.78)
1:16	46	42	91.3	79.1–97.1	4	8.7	6.72–24.59 (20.39)
1:32	43	43	100	NA^*a*^	0	0	17.96–25.09 (22.18)
1:64	41	40	97.6	86.3–99.9	1	2.4	8.66–26.32 (22.53)
1:128	40	40	100	NA	0	0	19.82–27.59 (23.29)
1:256	32	32	100	NA	0	0	19.71–26.25 (23.10)
1:512	19	19	100	NA	0	0	21.37–26.5 (23.70)
>1:512	21	21	100	NA	0	0	20.35–33.68 (24.12)
	450	343	76.2	72.1–79.9	107	23.8	
Pregnant subgroup							
Not reactive	37	2	5.4	0.5–18.6	35	94.6	1.19–17.44
1:1	10	4	40	16.7–68.8	6	60	9.61–18.85
1:2	13	6	46.2	23.9–70.9	7	53.8	9.11–20.6
1:4	10	6	60	31.2–83.2	4	40	7.90–21.67
1:8	5	5	100	NA	0	0	16.11–22.87
1:16	4	4	100	NA	0	0	12.33–20.45
1:32	2	2	100	NA	0	0	21.01; 22.38
1:64	2	2	100	NA	0	0	20.82; 21.14
1:128	4	4	100	NA	0	0	20.51–27.59
1:256	1	1	100	NA	0	0	22.05
1:512	1	1	100	NA	0	0	22.57
	89	37	41.6	31.9–51.9	52	58.4	

^*a*^ NA indicates not applicable.

Samples with non-reactive RPR titers, which were also CMIA reactive, most likely represent past treated or early infections, and in this category, just 5 (7.6%) positive INSTI multiplex results were recorded, and the majority were negative. For pregnant females, 5.4% of the samples tested positive in the rapid test, which is similar to the percentage (7.6%) for the non-pregnant group with a corresponding non-reactive RPR titer. We noted that as the RPR titer increases, so does the percentage of positive INSTI multiplex results, that is, from 33.3% for samples with an RPR titer of 1:1 to 100% when the RPR titer is equal to or greater than 1:128. At a titer of 1:8, the sensitivity of the rapid test markedly increased to 93.8% compared with 83.3%% at the preceding titer of 1:4, also reflected in the confidence intervals between these two RPR titer categories. The diagnostic accuracy of the rapid test at RPR titers of 1:8 and greater was 97.2% (282/290). The overall sensitivity of the INSTI multiplex device for any reactive RPR titer, that is, from 1:1 to >1:512, was 88.02% (338/384; 95% CI: 84.4%–90.9%). A similar trend was noted in the pregnant female subgroup (Table 2), although at a RPR titer of 1:8, all of the rapid tests were positive, compared with 93.8% for the non-pregnant group. Additionally, the percentage of positive results for any reactive RPR titer was 67.3% (35/52; 95% CI: 53.7%–78.5%) versus 88.02% for the non-pregnant sample group.

There were eight samples with RPR titers of 1:8, 1:16, and 1:64 that repeatedly tested negative in the INSTI multiplex device. Consequently, we repeated the RPR assay to confirm that the previously recorded titer was within a 2-fold titer range, and in all these cases, we reconfirmed the original RPR titers to be as initially recorded.

Included in the panel were paired samples from 12 individuals, collected at two different time points, likely related either to post-treatment monitoring or, as in one case, a suspected reinfection. Five of these individuals, including a pregnant female, showed a 4-fold difference in RPR titers within a 6-month period, illustrated in Table 3, whereas the other seven patients had high but stable RPR titers in the same timeframe and are therefore not included in the Table. Patient 1 was a suspect reinfection based upon the RPR titer change that was not reactive in the rapid test on the initial serum collected in January, later increasing to a titer of 1:256 in April that yielded a positive rapid result. In addition, these paired samples showed an increase in the S/CO ratios, reflective of the increase in treponemal antibody levels. The other four patients all showed 4-fold or greater RPR titer reductions, one of whom (Patient 3) went from 1:256 in May to a not reactive titer in October of that year, although the rapid test still yielded a positive result on the RPR-negative baseline blood. Interestingly, the RPR not reactive result on these two patients (#1 and #3) had similar S/CO ratios (15.1 and 15.21, respectively), although one gave a negative rapid result, and the other was positive. Further characterization from the clinical history was not possible due to limitations placed on access to the patient’s charts. The RPR titers from the paired samples of the other seven patients were all greater than 1:8, and not surprisingly, all of them yielded a positive INSTI multiplex result, and additionally, the S/CO ratios were not very different between the samples collected at different time points.

**TABLE 3:** Comparison of RPR titers, INSTI multiplex results, and S/CO ratios for paired samples from selected patients

Patient no.	Blood collection time	RPR titer	S/CO ratio	INSTI result
Patient 1	Jan 2023	Not reactive	15.1	Negative
	Apr 2023	1:256	24.9	Positive
Patient 2	Nov 2022	1:256	21.16	Positive
	Mar 2023	1:16	20.99	Positive
Patient 3	May 2022	1:256	19.79	Positive
	Oct 2022	Not reactive	15.21	Positive
Patient 4^*a*^	Nov 2022	1:128	20.51	Positive
	Apr 2023	1:16	21.17	Positive
Patient 5	Jan 2023	1:512	24.80	Positive
	Mar 2023	1:32	25.03	Positive

^*a*^ Pregnant female.

**Fig 1:**
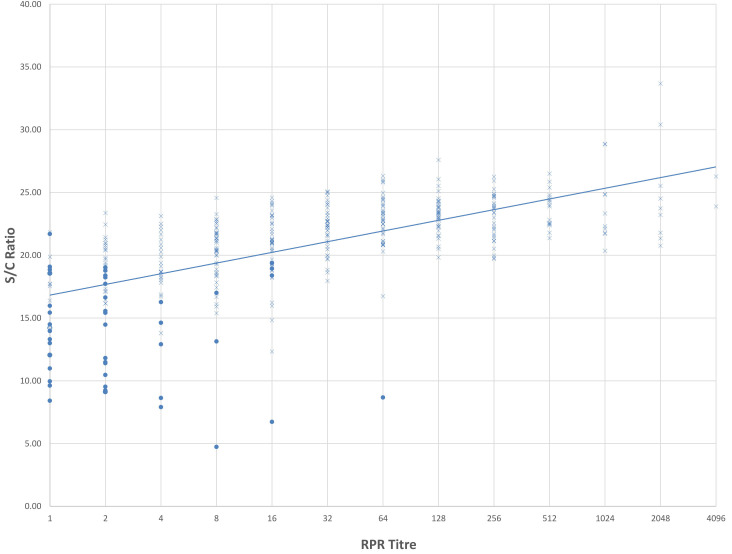
Comparison of positive and negative rapid test results by RPR titer (1:1 and greater) and S/CO value. Positive rapid results are denoted by a cross and negative results by a filled circle.

Figure 1 illustrates the S/CO ratios of samples, with their respective RPR titer and INSTI multiplex test result, as individual plots derived from the data listed in Table 2, restricted to samples with RPR titers of 1:1 and upward. Positive rapid results are plotted as an X, whereas negative results are filled dots due to their much lower frequency and make these data points more obvious. At the lower RPR titers of 1:1 and 1:2, most of the negative rapid test result S/C ratios are below the trendline, whereas at titers of 1:4 and above, the numbers of positive rapid results are most often above the trendline.

In Table 2, the mean of the S/CO ratios at each RPR titer category shows an incremental increase, with the lowest S/CO ratio (9.60) recorded for the samples with no RPR titer. As well, there is a marked difference in the mean S/CO ratios between not reactive RPR titer and the RPR titer of 1:1 (9.6 vs 16.56, respectively), whereas for the remainder of the RPR titer categories, the mean S/CO ratio differences vary from 0.14 to 1.69 between them and tend to be much smaller at the upper RPR titers of >1:512.

For 66 CMIA-reactive samples with no RPR titer, five yielded a positive rapid result, and two of these were from pregnant females. The S/CO ratios for these positive samples ranged from 13.73 to 18.32, which is also within the ranges of values of sera with RPR titers of 1:8 and greater that consistently gave positive rapid test results. Furthermore, in this category, there were four samples with S/CO ratios ranging from 15.15 to 18.32 that gave negative rapid results, which would be expected to yield a positive rapid result based upon the mean baseline S/CO ratios noted in the RPR categories of 1:8 and upward.

Likewise for the pregnant female categories, at RPR titers of 1:8 and greater, all the INSTI multiplex samples gave a positive result, whereas for the larger panel, a few samples tested negative. In part, the explanation could be ascribed to the lowest S/CO ratio recorded at 16.11 for the pregnant females, which is in the range of the S/CO ratios from the larger panel that were consistently positive in the rapid test. However, the numbers in these categories were much smaller than the larger panel, making it difficult to draw firm conclusions from this observation.

There were eight samples with RPR titers of 1:8, 1:16, and 1:64, which yielded a negative rapid test result when a positive rapid result would be expected. Inspection of their respective S/CO ratios, shown as filled dots in Fig. 1, all placed well below the median ranges for these titers and well below the trendline.

In summary, a general observation is that as the RPR titer increases, there is a trend toward the mean S/CO ratio also increasing, although at RPR titers of 1:1024 and above, S/CO ratios tend to plateau and are mainly below the trendline shown in Fig. 1.

## DISCUSSION

A larger evaluation of the INSTI multiplex kit using a structured serum panel, comprising 900 negative (CMIA S/CO ratio <1.0) and 450 positives (CMIA S/CO ratio >1.0), was performed here to supplement the results obtained from the PoSH study completed in 2022 by Singh et al*.* (15), with a view to implementing a decentralized testing program in acute care facilities located in catchment areas with high rates of infectious syphilis and congenital infections in Alberta. Most of the larger acute care facilities have a 24/7 laboratory providing STAT services across several disciplines that would make it possible to offer rapid syphilis testing while the individual is either an inpatient or awaiting assessment in the Emergency Department.

In the PoSH study, 1,364 samples were evaluated using fingerstick blood for the INSTI multiplex test, and serum was collected for comparative standard syphilis serology, of which 489 samples were found to be CMIA reactive, with and without RPR titers. As noted in both the PoSH study and this evaluation, syphilis-reactive samples with a negative RPR titer were very likely to test negative in the rapid syphilis assay, 28.4% in the PoSH study versus 7.6% for this panel. As in the PoSH study, panel sera with RPR titers from 1:1 to 1:4 were more likely to test positive, with the sensitivity increasing from 33% to 83.3% (average 59.6%) compared with 78.6% in the PoSH study. The reasons for the larger differences in sensitivity between the serum panel and PoSH study may arise from the possibility that the panel samples were selected from treated cases and hence more likely to test negative, whereas in the PoSH study, these cases were more likely to be untreated at the time of testing. As well, fingerstick blood was tested at the outreach site or clinic location and not on the parallel blood sample collected for standard serology comparison. Therefore, the results from this study panel contribute to the performance of this assay for serum, the sample of choice for syphilis serological investigations in laboratory settings. Our study also reaffirms the findings of the PoSH study showing a high correlation between a positive rapid serum result with an RPR titer of 1:8 and greater, with a sensitivity of at least 93.8% and PPV of 99.1%, therefore a positive rapid result indicates an active syphilis infection. Furthermore, cases with high RPR titers were from individuals in the primary, secondary, or early latent phases, based on later staging, which would likely apply to the panel samples with similar RPR titers.

We also investigated the potential of a false-positive rapid result, especially in the pregnant female subgroup of 180 CMIA-negative samples, and did not find any. False-positive serology results, which may result in unnecessary treatment, are infrequently encountered in this population when using the reverse sequence algorithm to screen for syphilis (19).

Another finding from the PoSH study was the number of invalid results reported from the two point-of-care devices when using a fingerstick blood sample, although the numbers were significantly lower for the INSTI multiplex POCT that we evaluated in this study. Invalid results can often arise from insufficient sample being tested, the residual background color of lysed blood coloring the membrane, therefore making the control dot difficult to discern visually, or low levels of antibody present in persons with agammaglobulinaemia not triggering the internal IgM/IgG control. We encountered no invalid results with this serum panel, as serum is a much cleaner sample type with less background than a whole blood or fingerstick blood sample. Therefore, in laboratory settings where centrifuges are commonplace, the preference would be to use serum over whole blood when running these samples as STAT or urgent tests, which would minimally add to the processing time. We estimate that the time interval from receipt of a sample at the laboratory to a result is easily feasible within an hour or thereabouts.

A point of consideration is that serology point-of-care devices are usually regarded as screening assays, and confirmatory testing is recommended for positive results (20), which again would add to the delay to a final result and potentially treatment initiation. However, from the combined findings of this evaluation and the PoSH study, the likelihood of a false-positive finding resulting in overtreatment is <0.5%. Therefore, cases without prior syphilis or previously negative results can be treated immediately with a high level of confidence due to the high accuracy of this rapid test.

We examined the association of the RPR titer and S/CO ratio of the syphilis CMIA to determine if there is a S/CO ratio cutoff that would reliably separate a positive from a negative rapid result. From the Abbott CMIA package insert (21), the syphilis antigen(s) are treponemal recombinant proteins to TpN15, TpN17, and TpN47, two of which, namely TpN15 and TpN17, are similar to those used in the INSTI multiplex kit. Furthermore, the manufacturer of the syphilis CMIA states that there is a direct relationship between the amount of anti-treponemal antibody measured in the sample to the S/CO ratio. This association was previously demonstrated in a study by Dai et al*.* (22) where they showed a positive correlation of S/CO ratios with TPPA titers in a prospective study of nearly 9,000 samples in order to determine a S/CO ratio cutoff where the confirmatory TPPA result would be expected to be reliably positive. Although they also performed the TRUST (Toluidine Red Unheated Serum Test) non-treponemal assay on the reactive samples as an additional confirmatory test, they noted that just 40% were reactive but unfortunately did not provide the TRUST titers in their publication. However, they did find that TRUST-positive samples were more likely to have an S/CO ratio of 9.9 and greater, which is similar to our findings. Therefore, in the early stages of syphilis, it is not unreasonable to expect rising amounts of treponemal antibodies, which, in turn, parallel the rising non-treponemal RPR titers. In contrast, treated patients represented by the CMIA-reactive, but RPR non-reactive titers in our study, have lower treponemal antibody levels, as reflected in the lower mean S/CO value (9.60) and are in turn also associated with a negative rapid syphilis result. In the categories with lower RPR titers, namely 1:1–1:4, these samples may represent either treated patients with declining treponemal antibody levels or early infections with rising titers, hence the finding of both positive and negative rapid results.

In the study panel, we had paired samples from four cases that likely represented post-treatment declines in RPR titers, although the baseline titers were all above 1:8, and not unexpectedly, they all tested positive in the INSTI multiplex. This observation would suggest that post-treatment monitoring would still require the use of RPR titers in a laboratory setting. A publication by Gao et al*.* (9), using a rabbit model, showed that the non-treponemal or “reagin” antibodies produced by the infected host arise from a robust immune response to both the damaged host-cardiolipin and *T. pallidum* cardiolipin antigen located within the inner membrane of this spirochete. However, the syphilis antigen component in this rapid kit is based upon the treponemal surface membranes of the organism, and historically treponemal antibodies have not been used to monitor for post-treatment effectiveness as they continue to be present. We clearly found that the majority of samples with no RPR titers still had some residual treponemal antibody based upon the CMIA and TPPA tests, which tested negative in this INSTI multiplex device, which is designed to detect treponemal antibodies. Consequently, although residual treponemal antibodies remain after treatment when RPR titers have returned to baseline or are low, there is a potential opportunity to study reinfections in treated cases, as it would not be unreasonable to expect that the treponemal antibodies would also show a marked increase in response to reinfection.

Serological testing for syphilis in Alberta is performed at centralized laboratory locations, as in many other provinces and states in Canada, the USA, and other developed countries in the world. Although these high-throughput platforms can screen significantly more samples for treponemal antibodies that are detectable early in the course of the infection rather than first testing for non-treponemal antibodies using the manual rapid plasma reagin card test, the time to a result can vary from 1 to 3 days for a negative to 5 days or more for a positive from collection of the blood to reporting a result. When the additional time to notify and treat a patient is factored in, there are ample opportunities for the index case to transmit syphilis to additional sexual contacts, thus perpetuating the outbreak cycle. A recent Alberta study (23) reviewing care milestones to prevent syphilis transmission in pregnant females and congenital infections noted that in their data set of 182 infected pregnant women, 98.9% had a healthcare encounter at some point during their pregnancy, but just 39% were screened for syphilis. Therefore, the availability of rapid testing in acute care facilities in regions where high rates of congenital infections and infectious syphilis are recorded presents yet another opportunity to detect and treat potential cases immediately, which is not possible with current laboratory specialty centralization practices and resulting turnaround times.

Immediate treatment can prevent onward transmission and limit disease progression, and in uncomplicated cases of infectious syphilis, a single dose of benzathine penicillin G [2.4 million units (intramuscularly)] is effective in treating the infection and preventing ongoing transmission (24, 25). Furthermore, a positive rapid result from a pregnant female provides a compelling reason to urgently initiate linkage to specialist care, especially when both the mother and baby require a longer treatment schedule together with regular follow-ups to ensure that the infection is adequately treated in both patients and subsequently to monitor the development milestones of the baby.

Two limitations of this study are as follows: first, we did not look at the performance of the HIV component of this device, in part, as there is an HIV-only assay from this company that has been licensed for a few years both in Canada and the USA, which uses the same antigen and antibody detection components used in this newer multiplex device we evaluated here. Furthermore, current laboratory guidelines are to confirm all screen HIV serology-positive results with confirmatory tests, whether these are from POCT or standard fourth-generation assays, to exclude false positives that occasionally occur with the screening tests, before initiating anti-retroviral treatment (26, 27). Second, staging was not easily available for the CMIA-positive samples in this panel, which may have clarified some of the negative rapid results with high RPR titers. However, we considered that the combined findings from the PoSH study and our evaluation would provide contemporary and complementary data on the performance of the assay for serum samples in a laboratory-based approach to providing local rapid testing in outbreak-affected centers.

The strength of this evaluation was the use of a broader panel of sera populated with samples with characterized RPR titers over a wide range, supplemented with S/CO ratio data that further provide a measure of treponemal antibody levels in conjunction with a positive or negative rapid result. Additionally, the selection of serum samples comprising the panel was by age categories reflective of the demographics of the patient population in the current syphilis outbreak in Alberta (28). Although the validation was performed by a single technologist, the rationale was to provide consistency and familiarity in performing, reading, and interpreting the rapid result. However, we acknowledge that this is a potential limitation where the use of this rapid device in wider laboratory settings could demonstrate much more variability with multiple users performing this test. Such a limitation can be countered by providing training and validation panels, reference material, and ongoing quality assurance panels with “difficult samples” to help achieve consistent interpretation and reporting. Also, the use of serum or plasma as the preferred sample type compared with whole blood also assists with a cleaner reading of the test and control dots without the residual hemoglobin background when using whole blood.

In summary, our study illustrates the high performance of the INSTI Multiplex HIV-1/2 Syphilis kit using a large panel of well-characterized serum samples. As a syphilis result is available within minutes, this test could be included in acute care STAT laboratory menus to enable single visit testing and treatment at various patient encounters for inpatients, labor and delivery units, and emergency departments.
